# Hyperuricemia remission after colorectal cancer surgery for colorectal cancer patients

**DOI:** 10.1038/s41598-023-46348-w

**Published:** 2023-11-01

**Authors:** Fei Liu, Yin Huang, Zi-Wei Li, Xu-Rui Liu, Xiao-Yu Liu, Quan Lv, Xin-Peng Shu, Lian-Shuo Li, Wei Zhang, Yue Tong, Meng-Hua Zeng, Dong Peng

**Affiliations:** Department of Gastrointestinal Surgery, The First Affiliated Hospital of Chongqing Medical University, Chongqing, 400016 China

**Keywords:** Cancer, Risk factors

## Abstract

The purpose of this study was to investigate whether patients with colorectal cancer (CRC) combined with hyperuricemia remitted 1 year after CRC surgery. CRC patients combined with hyperuricemia who underwent radical surgery were included from a single clinical center from Jan 2016 to Dec 2021. Baseline characteristics was compared between the remission group and the non-remission group. Multivariate logistic regression was used to find the possible predictive factors of hyperuricemia remission. A total of 91 patients were included for data analysis, retrospectively. There were 34 (37.4%) patients in the remission group and 57 (62.6%) patients in the non-remission group. The mean preoperative weight and body mass index (BMI) were 61.2 ± 10.7 (kg) and 24.1 ± 3.3 (kg/m^2^). 21 (23.1%) patients had a history of drinking. We found that the weight and BMI were not significantly different before and 1 year after CRC surgery (*P* > 0.05). In contrast, uric acid values were significantly decreased (*P* < 0.01). Meanwhile, the outcomes showed there were no significant differences in the baseline characteristics between the remission and non-remission groups (*P* > 0.05). According to multivariate logistic regression, we found that the history of drinking was a predictive factor of hyperuricemia remission (OR = 0.046, 95% CI 0.005–0.475, *P* = 0.010). CRC patients with hyperuricemia had a 37.4% remission from hyperuricemia 1 year after CRC surgery. Tumor location, tumor stage, and tumor size did not predict the remission of hyperuricemia. Notably, the history of drinking was a predictive factor of hyperuricemia remission.

## Introduction

Colorectal cancer (CRC) is the most common cancer of the digestive system and the third most common cancer in the world^1,2^. Approximately 35,000 new CRC cases are diagnosed each year, accounting for 20% of all cancer diagnoses^2,3^. CRC is also the second most death-related cancer in the world, with approximately 16,000 cancer-related deaths each year^2^. At present, surgery is the most effective means of treating CRC^4,5^.

Uric acid (UA) is the product of purine metabolism in all cells. UA is produced by the oxidation of xanthine and hypoxanthine through xanthine oxidoreductase, which is a necessary reaction to remove nitrogenous waste products from the body^6^. UA values in normal individuals range from 178 to 400 (μmol/L)^7^. Hyperuricemia is caused by the abnormal UA metabolism, including the urate overproduction and decreased UA excretion^8,9^. In recent years, the prevalence of hyperuricemia in developing countries has been rising^10,11^. Studies have shown that hyperuricemia may be associated with increased predictive of cancer incidence and metastasis and is associated with the risk of cancer death^12,13^.

Notably, a previous study suggests that patients with concurrent CRC and type 2 diabetes mellitus (T2DM) have a remission 1 year after CRC surgery^14^. Recent evidence has demonstrated that patients with concurrent CRC and hypertension had a remission rate 1 year after CRC surgery as well^3^. Hypertension, T2DM, and hyperuricemia are both forms of metabolic syndrome. However, there is no report showing whether patients with concurrent CRC and hyperuricemia have a remission after CRC surgery. Therefore, this study is to investigate whether patients with concurrent CRC and hyperuricemia remitted 1 year after CRC surgery.

## Method

### Patients

This retrospective study enrolled 91 CRC patients combined with hyperuricemia and underwent surgery from a single clinical center from Jan 2016 to Dec 2021. This study was approved by the Institutional Ethics Committee of the First Affiliated Hospital of Chongqing Medical University (2022-133-2).

### Inclusion and exclusion criteria

The CRC patients combined with hyperuricemia who underwent radical surgery were included from a single clinical center (n = 270). The exclusion criteria were as follows: 1, tumor stage IV patients (n = 3); 2, incomplete post-operative information (n = 94); 3, unreachable patients (n = 82). Finally, 91 patients were included for data analysis.

### Data collection

We obtained preoperative baseline information and postoperative recovery of the patient through electronic medical records and telephone follow-up. The preoperative information included age, sex, hypertension, T2DM, weight, body mass index (BMI), smoking, drinking, albumin, hemoglobin, tumor location, tumor nodes metastasis (TNM) stage, and tumor size. As for postoperative information, weight, BMI, and UA were collected.

#### Definition

The tumor node metastasis stage was diagnosed according to the AJCC 8th Edition ^15^. The remission group was defined as a return to normal UA values or a significant decrease in postoperative gout compared to preoperative levels by 1 year after CRC surgery. Weight change was defined as the postoperative weight minus preoperative weight.

### Statistical analysis

Continuous variables were expressed as mean ± standard deviation (SD). Categorical variables were expressed as n (%). The Chi-square test was used to analyze categorical variables and the t-test was used to analyze continuous variables between the remission group and the non-remission group. Paired-sample t-test was used to analyze continuous variables before and 1 year after surgery. Statistical analysis was performed by the SPSS (version 22.0) software. *P*-value of < 0.05 was considered statistically significant.

### Ethics approval and informed consent

The study was approved by the ethics committee of our institution (The First Affiliated Hospital of Chongqing Medical University, 2022-133-2), and all patients signed informed consent. This study was conducted in accordance with the World Medical Association Declaration of Helsinki as well.

## Results

### Patients

A total of 91 patients were included in this study according to the inclusion and exclusion criteria. The age of included patients was 63.7 ± 11.5 years old. There were 31 (34.1%) males and 60 (65.9%) females. The mean preoperative weight and BMI were 61.2 ± 10.7 (kg) and 24.1 ± 3.3 (kg/m^2^). 24 (26.4%) patients had a history of smoking, and 21 (23.1%) patients had a history of drinking. In addition, more details including hypertension, T2DM, albumin, hemoglobin, tumor location, TNM stage, and tumor size were shown in Table 1.

**Table 1:** Baseline characteristics of patients with concurrent CRC and hyperuricemia.

Characteristics	N = 91
Age, year	63.7 ± 11.5
Sex	
Male	31 (34.1%)
Female	60 (65.9%)
Hypertension	43 (47.3%)
T2DM	8 (8.8%)
Weight preoperative, kg	61.2 ± 10.7
BMI preoperative, kg/m^2^	24.1 ± 3.3
Smoking	24 (26.4%)
Drinking	21 (23.1%)
Albumin, g/L	41.2 ± 4.7
Hemoglobin, g/L	124.5 ± 21.8
Tumor location	
Colon	38 (41.8%)
Rectum	53 (58.2%)
TNM stage	
I	25 (27.5%)
II	42 (46.2%)
III	24 (26.4%)
Tumor size	
< 5 cm	63 (69.2%)
≥ 5cm	28 (30.8%)

Variables are expressed as the mean ± SD or n (%).

CRC, colorectal cancer; T2DM, type 2 diabetes mellitus; BMI, body mass index; TNM, tumor nodes metastasis.

### Comparison before and 1 year after CRC surgery

Table 2 showed the changes of weight, BMI, and UA before and 1 year after CRC surgery. As Table 2 suggested, the UA after CRC surgery (522.3 ± 57.8, umol/L) was significantly lower than the UA before CRC surgery (428.1 ± 81.7, umol/L) (*P* < 0.01). However, there were no significant differences in weight and BMI before and 1 year after CRC surgery (*P* > 0.05).

**Table 2:** Comparison before and one year after CRC surgery (N = 91).

Characteristics	Before	After	*P*
Weight, kg	61.2 ± 10.7	62.4 ± 10.3	0.096
BMI, kg/m^2^	24.1 ± 3.3	24.6 ± 3.3	0.089
Uric acid, umol/L	522.3 ± 57.8	428.1 ± 81.7	< 0.01*

Variables are expressed as the mean ± SD, *P-value < 0.05.

CRC, colorectal cancer.

### Baseline characteristics between the remission and non-remission groups

Patients were divided into the remission group and the non-remission group. There were 34 (37.4%) patients in the remission group and 57 (62.6%) patients in the non-remission group. We compared the baseline characteristics including age, sex, hypertension, T2DM, weight, BMI, smoking, drinking, albumin, hemoglobin, tumor location, TNM stage, and tumor size between the remission and non-remission groups. After data analysis, the outcomes showed there were no significant differences in the baseline characteristics between the remission and non-remission groups (*P* > 0.05). (Table 3).

**Table 3:** Baseline characteristics of the remission group and the non-remission group.

Characteristics	Remission (34)	Non-remission (57)	*P* value
Age, year	63.0 ± 12.9	64.1 ± 10.7	0.646
Sex			0.849
Male	12 (35.3%)	19 (33.3%)	
Female	22 (64.7%)	38 (66.7%)	
Hypertension	17 (50.0%)	26 (45.6%)	0.685
T2DM	2 (5.9%)	6 (10.5%)	0.705
Weight preoperative, kg	61.9 ± 11.5	60.9 ± 10.2	0.671
BMI preoperative, kg/m^2^	23.8 ± 3.9	24.2 ± 3.0	0.582
Smoking	9 (26.5%)	15 (26.3%)	0.987
Drinking	5 (14.7%)	16 (28.1%)	0.143
Albumin, g/L	41.0 ± 4.3	41.3 ± 4.9	0.766
Hemoglobin, g/L	124.9 ± 24.3	124.2 ± 20.3	0.888
Tumor location			0.218
Colon	17 (50.0%)	21 (36.8%)	
Rectum	17 (50.0%)	36 (63.2%)	
Tumor stage			
I	7 (20.6%)	18 (31.6%)	0.431
II	16 (47.1%)	16 (28.1%)	
III	11 (32.4%)	11 (19.3%)	
Tumor size			0.828
< 5 cm	24 (70.6%)	39 (68.4%)	
≥ 5 cm	10 (29.4%)	18 (31.6%)	

Variables are expressed as the mean ± SD, n (%), *P-value < 0.05.

CRC, colorectal cancer; T2DM, type 2 diabetes mellitus; BMI, body mass index.

### Multivariate logistic regression of hyperuricemia remission

Multivariate logistic regression was used to find the possible predictive factors of hyperuricemia remission. Pre-operative weight, weight change, drinking, and tumor stage were collected for multivariate logistic regression analysis. After data analysis, we found that the history of drinking was a predictive factor of hyperuricemia remission (OR = 0.272, 95% CI 0.076–0.975, *P* = 0.046). However, pre-operative weight (OR = 1.030, 95% CI 0.979–1.083, *P* = 0.251), weight change (OR = 1.016, 95% CI 0.947–1.090, *P* = 0.661), and tumor stage (OR = 1.629, 95% CI 0.880–3.018, *P* = 0.121) were not associated with hyperuricemia remission. (Table 4).

**Table 4:** Multivariate logistic regression of hyperuricemia remission.

Risk factors	OR (95% CI)	*P* value
Pre-operative weight (kg)	1.030 (0.979–1.083)	0.251
Weight change (kg)	1.016 (0.947–1.090)	0.661
Drinking (yes/ no)	0.272 (0.076–0.975)	0.046*
Tumor stage (III/II/I)	1.629 (0.880–3.018)	0.121

*P-value < 0.05.

OR, odds ratio; CI, confidence interval.

## Discussion

We enrolled 91 CRC patients with hyperuricemia who underwent surgery for analysis. There were 34 patients in the remission group, and the remission rate was 37.4%. After data analysis, there were no significant differences in the baseline characteristics including age, sex, hypertension, T2DM, weight, BMI, smoking, drinking, albumin, hemoglobin, tumor location, TNM stage, and tumor size between the remission and non-remission groups. However, we found that less preoperative drinking was a predictor of remission of postoperative hyperuricemia in the multivariate logistic regression.

UA was the product of nucleotide metabolism and was produced by the liver, muscle, and intestine^16^. High levels of UA were often observed in patients with metabolic syndrome. Some studies reported that high levels of UA might contribute to the onset and development of metabolic syndrome^17,18^. Hyperuricemia was partly responsible for insulin resistance, which was a key factor in the metabolic syndrome. Furthermore, Hyperinsulinemia due to insulin resistance could impair the excretory function of the kidneys and led to hyperuricemia^19^. Thus, there might be a bidirectional causal relationship between hyperuricemia and insulin resistance. Insulin resistance was a cause of T2DM. Both hyperuricemia and T2DM were metabolic syndromes. Metabolic disorders were quite associated with central obesity, insulin resistance, hypertension and hyperlipemia in previous studies^20–22^.

In previous study, Peng et al.^14^. in a retrospective study of 296 patients with CRC with T2DM found a remission 1 year after surgery. Moreover, they also reported that higher weight loss and shorter duration of T2DM contributed to T2DM remission. Furthermore, Chen et al. ^3^ found patients with concurrent CRC and hypertension had a remission rate 1 year after CRC surgery as well through analysis of 387 patients. As public known, hypertension, type 2 diabetes and hyperuricemia were all associated with metabolic disorders. Theoretically, hyperuricemia could also be alleviated after CRC surgery. However, there were no studies reported whether CRC patients with hyperuricemia had a remission 1 year after surgery. Therefore, it was necessary to investigate whether hyperuricemia in patients with CRC combined with hyperuricemia remitted 1 year after CRC surgery and potential predictive factors for remission.

In this study, we found a 37.4% remission rate of hyperuricemia one after CRC surgery. Furthermore, patients who drank less could contribute hyperuricemia remission. The mechanisms were not yet clear, but the reasons might be as follows: First, patients’ dietary structure changed after surgery. There were some studies suggesting a correlation existed between the composition of the diet and hyperuricemia^23–25^. Meat and poultry could contribute to elevated UA^26^. Second, patients reduced alcohol consumption. Alcohol was a recognized group of carcinogens, and its dangers were self-evident. Men who were currently drinking alcohol were at higher risk of developing hyperuricemia, and drinking was a risk factor for hyperuricemia^27–29^. In general, patients would reduce or even refuse to drink after surgery due to increased awareness of the dangers of alcohol. Third, with resection of the primary tumor, correction of endocrine dysregulation, reduction of the pro-inflammatory response or remodeling of the tumor microenvironment might result in a decrease in UA. Forth, previous studies found that remission of hypertension or T2DM might be related to hormones, including renin, angiotensin, aldosterone, ghrelin, glucagon-like peptide (GLP)-1 and GLP-2^3,14,30,31^. This reason might also bring relief from hyperuricemia, which was also a metabolic disease. Uric acid was a dynamic parameter and was influenced by diverse factors, such as age^32^, sex^27^, location^33^, smoking^34,35^, gout, thiaside use, and increased cell cycle situations. There was a lack of knowledge about these factors. The patient might have changed his or her lifestyle after the surgery, resulting in a change in uric acid.

To our knowledge, this was the first study to investigate whether hyperuricemia in patients with CRC combined with hyperuricemia remitted 1 year after CRC surgery and potential predictive factors for remission. The baseline characteristics were not significantly different between the remission group and the non-remission group, which could make our outcomes convincing. However, there were still some limitations. First, the sample size of this study was small. Second, the patients in this study were from a single clinic center, which might cause selected bias. Thus, more research should be conducted in the future.

In conclusion, CRC patients with hyperuricemia had a 37.4% remission from hyperuricemia 1 year after CRC surgery. Tumor location, tumor stage, and tumor size did not predict the remission of hyperuricemia. Notably, the history of drinking was a predictive factor of hyperuricemia remission.

## Data Availability

The datasets used and analyzed during the current study are available from the corresponding author on reasonable request.
